# Cancer-Net SCa: tailored deep neural network designs for detection of skin cancer from dermoscopy images

**DOI:** 10.1186/s12880-022-00871-w

**Published:** 2022-08-09

**Authors:** James Ren Hou Lee, Maya Pavlova, Mahmoud Famouri, Alexander Wong

**Affiliations:** 1 Vision and Image Processing Research Group, University of Waterloo, Waterloo, Canada; 2 Waterloo Artificial Intelligence Institute, University of Waterloo, Waterloo, Canada; 3 DarwinAI Corp, Waterloo, Canada

**Keywords:** Akin cancer, Melanoma, Deep neural network, Self-attention

## Abstract

**Background:**

Skin cancer continues to be the most frequently diagnosed form of cancer in the U.S., with not only significant effects on health and well-being but also significant economic costs associated with treatment. A crucial step to the treatment and management of skin cancer is effective early detection with key screening approaches such as dermoscopy examinations, leading to stronger recovery prognoses. Motivated by the advances of deep learning and inspired by the open source initiatives in the research community, in this study we introduce Cancer-Net SCa, a suite of deep neural network designs tailored for the detection of skin cancer from dermoscopy images that is open source and available to the general public. To the best of the authors’ knowledge, Cancer-Net SCa comprises the first machine-driven design of deep neural network architectures tailored specifically for skin cancer detection, one of which leverages attention condensers for an efficient self-attention design.

**Results:**

We investigate and audit the behaviour of Cancer-Net SCa in a responsible and transparent manner through explainability-driven performance validation. All the proposed designs achieved improved accuracy when compared to the ResNet-50 architecture while also achieving significantly reduced architectural and computational complexity. In addition, when evaluating the decision making process of the networks, it can be seen that diagnostically relevant critical factors are leveraged rather than irrelevant visual indicators and imaging artifacts.

**Conclusion:**

The proposed Cancer-Net SCa designs achieve strong skin cancer detection performance on the International Skin Imaging Collaboration (ISIC) dataset, while providing a strong balance between computation and architectural efficiency and accuracy. While Cancer-Net SCa is not a production-ready screening solution, the hope is that the release of Cancer-Net SCa in open source, open access form will encourage researchers, clinicians, and citizen data scientists alike to leverage and build upon them.

## Introduction

Skin cancer is the most frequently occurring form of cancer in the U.S., with over 5 million new cases diagnosed each year [1], and continues to rise with every year that passes. Furthermore, the annual cost of treating skin cancer in the U.S. alone is estimated to be over $8 billion [2]. Fortunately, prognosis is good for many forms of skin cancer when detected early [3], and as such early skin cancer detection is an important factor for patient recovery. This is particularly critical for melanoma, the deadliest form of skin cancer that, if left undiagnosed and untreated at an early stage, can spread beyond its original location to nearby skin and organs until surgery is no longer sufficient and treatment methods such as radiation are required [4]. As such, early diagnosis and preventative measures are exceedingly important as the death rate and cost of treatment both increase drastically as the cancer progresses from Stage I to Stage IV [5]. However, if diagnosed early on, a simple surgery to remove the lesion can increase survival rates by stopping the cancer from spreading beyond its origin [6, 7].

Currently, the most popular method of skin lesion diagnosis is the dermoscope assisted method [8], which is able to achieve a diagnostic accuracy of roughly 75–97% [9]. However, it was also found that in the hands of a dermatologist that has limited experience with the instrument, the use of a dermoscope may reduce the diagnostic accuracy rather than augmenting it. In addition, the “lack of reproducibility and subjectivity of human interpretation” [7] associated with human based diagnosis is one of the main reasons why there has been a significant increase in interest for computer assisted diagnosis of skin cancer. The use of computer vision and machine learning for the diagnosis of pigmented skin lesions has been shown to be accurate and practical [5–7, 9–13], and can improve biopsy decision making [10], as well as act as a pre-screening tool to reduce the amount of a time a professional spends on each case. Motivated by the challenge of skin cancer detection, and inspired by the open source and open access efforts of the research community, in this study we introduce **Cancer-Net SCa**, a suite of deep neural network designs tailored for the detection of skin cancer from dermoscopy images, one of which possesses a self-attention architecture design with attention condensers [14, 15]. To construct Cancer-Net SCa, we leveraged a machine-driven design strategy that leverages human experience and ingenuity with the meticulousness and raw speed of machines. We further leverage the International Skin Imaging Collaboration (ISIC) dataset [16] for this study, and illustrate the efficacy of Cancer-Net SCa when compared to previously proposed deep neural network architectures such as ResNet-50 [17] and Inception V3 [18], which were both leveraged in previous studies to great effect for skin cancer detection [19–21]. To the best of the authors’ knowledge, Cancer-Net SCa is comprised of the first machine-designed deep neural network architectures tailored specifically for skin cancer detection. Cancer-Net SCa is available to the general public in an open-source and open access manner. While Cancer-Net SCa is not a production-ready screening solution, the hope is that the release of Cancer-Net SCa will encourage researchers, clinicians, and citizen data scientists alike to leverage and build upon them.

The paper is organized as follows. We first discus related work on deep learning and machine learning methods as well as datasets for skin cancer detection. The next section describes the methodology leveraged to build the proposed Cancer-Net SCa, the overall network architecture designs of Cancer-Net SCa, as well as the explainability-driven performance validation strategy leveraged to audit Cancer-Net SCa. Following this, the next section presents the quantitative results for evaluating the efficacy of Cancer-Net SCa, qualitative results to gain insights into the decision-making behaviour of Cancer-Net SCa, and a discussion on the broader impact of methods such as Cancer-Net SCa for aiding the clinical decision process. Finally, conclusions are drawn and future work is discussed in the final section.

## Related work

Motivated by the tremendous advances in the field of deep learning [22] and the great potential it has shown in a wide range of clinical decision support applications [19, 23–29], a number of recent studies have explored the efficacy of deep neural networks for the purpose of skin cancer detection [19, 28, 30–37]. In a recent study by Budhiman et al. [19, 21], a comprehensive exploration of different residual network architectures was conducted for the purpose of melanoma detection, with the best quantitative results found when leveraging a ResNet-50 [38] architecture. Kassani and Kassani [35] performed a study on five different deep CNN architectures, and also determined that the ResNet50 architecture achieved the highest average F-score and accuracy when compared to networks leveraging AlexNet, VGGNet, and Xception based architectures. When comparing a ResNet-101 architecture against an Inception V3 architecture on a 2500 image subset of the ISIC dataset, similar performance was achieved in [20] with the Inception V3 architecture displaying an F-score that was 4% higher.

Alternative approaches to leveraging deep neural networks and others machine learning methods have been proposed for the task of melanoma detection from dermoscopy images. Codella et al. [39] demonstrate that an ensemble-driven technique leveraging a combination of hand-coded feature extractors, sparse-coding methods, SVMs, deep residual networks, and fully convolutional neural networks together can achieve state-of-the-art performance, with a sensitivity of 95% on the International Symposium on Biomedical Imaging (ISBI) 2016 dataset [40]. Hagerly et al. [41] also leverage deep learning with handcrafted features, and demonstrate that performance can be improved when using them in combination rather than individually. Recent efforts have also illustrated the effectiveness of attention based learning. Zhang et al. [42] leveraged the concept of attention residual learning to improve the ability to learn discriminating features by generating the attention weights from the classification trained network itself rather than from extra learnable layers. Evaluated on the ISIC-2017 dataset, the attention based networks in [42] achieved strong skin lesion classification performance in dermoscopy images. Yan et al. [43] also investigate visual attention based approaches for melanoma detection by introducing end-to-end trainable attention modules that are able to highlight relevant regions of the image, thus providing additional interpretable information to the end user. As such, the leveraging of deep learning for the task of skin cancer detection from dermoscopy images holds considerable promise. However, these more recent models tend to have a high architectural and computational complexity that do not work well in the context of edge or embedded devices. In order to integrate a deep learning solution into a portable dermoscopy device, or one that can be used in a dermatologist’s office, the model must be lightweight and efficient while maintaining a high performance. As such, a current problem that needs to be solved in this space is finding models that provide a good balance between accuracy and complexity in order to be leveraged for on-site dermatology assistance. In literature, models with fewer parameters have also been leveraged, with Chaturvedi et al. [44] using a MobileNet [45] architecture to classify the HAM10000 [46] dataset into seven classes of skin cancer. Taufiq et al. [47] and Castro et al. [48] have also created models designed for mobile devices, with the former using support vector machines and the latter using a CNN based on evolutionary algorithms. In this paper, we explore novel architectures that are also deployable on edge devices, catered towards usage in a fast-moving clinical environment.

In order for a deep neural network to be successfully built for skin cancer detection from dermoscopy images, large, high quality datasets are required. For the task of melanoma detection, most studies leverage the International Skin Imaging Collaboration (ISIC) [16] dataset, which is currently the largest curated skin lesion imaging dataset publicly available. However, other studies have also found success in leveraging alternative databases. Cıcero et al. [49] leveraged images downloaded from DermWeb [50], a digital atlas containing a list of dermatology related links. Ali et al. [51] created their own dataset through the use of Generative Adversarial Networks (GANs) with self-attention mechanisms, in order to generate realistic skin lesion samples to combat the frequent problem of unbalanced skin cancer datasets. As well, Hagerly et al. [41] used the HAM10000 [46] dataset in their study, a database containing over 9000 images labelled with five classes of skin cancer. Even with the abundance of quality skin images and datasets, the number of total samples is still orders of magnitude smaller than when compared to other domains, such as general image classification or text annotation. Labelling, segmentation, and acquisition of additional images is difficult, which leads many researchers to push for deep learning techniques when tackling this problem.

## Methods

### Data preparation

**Fig. 1:**
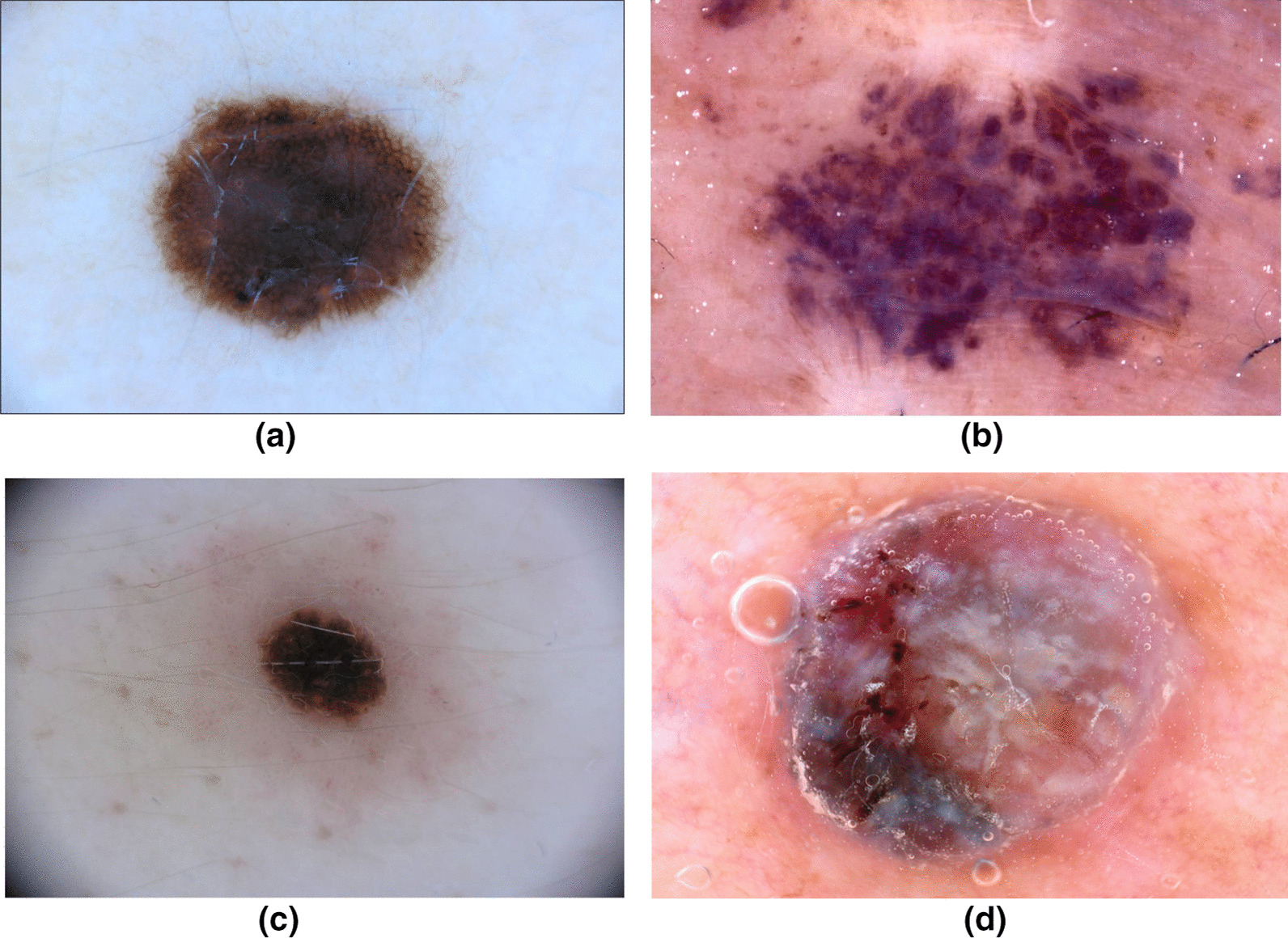
Sample images from the ISIC Dataset leveraged to build Cancer-Net SCa. Dermoscopy images **a** and **b** are both benign, while images **c** and **d** are both malignant. Image **b** can easily be mistaken for a malignant lesion, while image **c** can easily be misclassified as benign to the untrained eye

In this study, we leverage the International Skin Imaging Collaboration (ISIC) dataset, which is an open source public access archive of skin images. The dataset consists of 23,906 dermoscopy images at the time of the study, comprising a variety of skin cancer types such as Squamous Cell Carcinoma, Basal Cell Carcinoma, and Melanoma. A total of 21,660 dermoscopy images were labelled as either benign or malignant for Melanoma, composing the dataset leveraged to build Cancer-Net SCa. Out of these samples, 2286 were labelled as malignant, and the rest as benign. Example dermoscopy images from the ISIC dataset for both malignant and benign lesions are shown in Fig. 1. It can be observed from the example dermoscopy images that the problem of skin cancer detection from dermoscopy images is very challenging, with the benign lesion shown in Fig. 1b possessing morphological and textural heterogeneous characteristics commonly exhibited by malignant lesions, while the malignant lesion shown in Fig. 1c exhibiting morphological symmetry and textural uniformity commonly found in benign lesions. These examples motivate the exploration of deep neural networks for learning the subtle characteristics found in skin lesions as captured in dermoscopy images to better distinguish between benign and malignant lesions. Balanced random partitioning was conducted to form the training, validation, and test sets. Out of the 17,327 total samples in the training dataset, there were 1847 malignant samples. Out of the 2166 total samples in the validation dataset, there were 218 malignant samples. In contrast, the test dataset consisted of a balanced split of 221 benign samples and 221 malignant samples. This randomly sampled balanced split in testing enables for a better assessment of performance when compared with traditional k-fold cross validation, as k-fold performance can be skewed due to the major imbalances in the ISIC dataset.

During training, data augmentation was applied which included rotations (up to $30^{\circ }$), shifts (up to 10%), and vertical and horizontal flipping. Each image was resized to a size of $224\times 224$ pixels, with the 3-channel color information retained, with the exception of InceptionV3 [18], which took in images of shape $299\times 299\times 3$. Studies have shown that data augmentation methods such as illumination correction and contrast enhancement can improve image quality and generalization ability [35], and thus these augmentations are implemented as well ($\pm 10\%$ for either type). All methods were carried out in accordance with relevant guidelines and regulations.

### Machine-driven design exploration

To build the proposed Cancer-Net SCa deep neural network designs to be as tailored as possible around the task of skin cancer detection, we leveraged a machine-driven design exploration strategy to automatically design a highly customized deep neural network architecture based on characteristics of the ISIC dataset and the skin cancer detection task at hand. Leveraging this strategy allows for the automatic discovery of uniquely tailored macroarchitecture and microarchitecture designs that combine to achieve the optimal balance between representational power and complexity for skin cancer phenotype characterization from dermoscopy images, beyond what a hand-crafted deep neural network architecture can provide.

In this study, the machine-driven design exploration strategy we leveraged was generative synthesis [52], where the problem of identifying deep neural network architectures tailored for a specific task is formulated as a constrained optimization problem subject to a set of operational constraints related to the task and data at hand. More specifically, the constrained optimization problem posed here involves finding an optimal generator $\mathcal {G}$ whose generated deep neural network architectures $\left\{ N_s|s \in S\right\}$ maximize a universal performance function $\mathcal {U}$ (e.g., [53]), with constraints around operational requirements for a given task as defined by an indicator function $1_r(\cdot )$,

1 $$\begin{aligned} \mathcal {G} = \max_{\mathcal{G}}\,\mathcal{U}(\mathcal{G}(s))\quad\hbox{subject to}\quad 1_r(\mathcal{G}(s))=1,\quad \forall s \in S. \end{aligned}$$

where *S* represent a set of seeds to the generator. The approximate solution to the constrained optimization problem posed in Eq. 1 is determined via an iterative optimization process, with initiation of this optimization process based on an initial design prototype $\varphi$, $\mathcal {U}$, and $1_r(\cdot )$.

In this study, the operational constraint imposed within the indicator function $1_r(\cdot )$ was that the validation accuracy of the designed deep neural network exceeded that of the ResNet-50 architecture leveraged in [19], which was found by the authors of that study to provide the best quantitative results amongst different residual network architectures. For the initial design prototype $\varphi$, residual architecture design principles [17] were leveraged in this study. The use of residual connections have been shown to alleviate vanishing gradient and dimensionality problems, allowing networks to learn faster and easier with minor additional cost to architectural or computational complexity. Furthermore, given the iterative nature of the machine-driven design exploration strategy, we selected three of the generated deep neural network architecture designs to construct Cancer-Net SCa (i.e., Cancer-Net SCa-A, Cancer-Net SCa-B, and Cancer-Net SCa-C).

### Network architectures

**Fig. 2:**
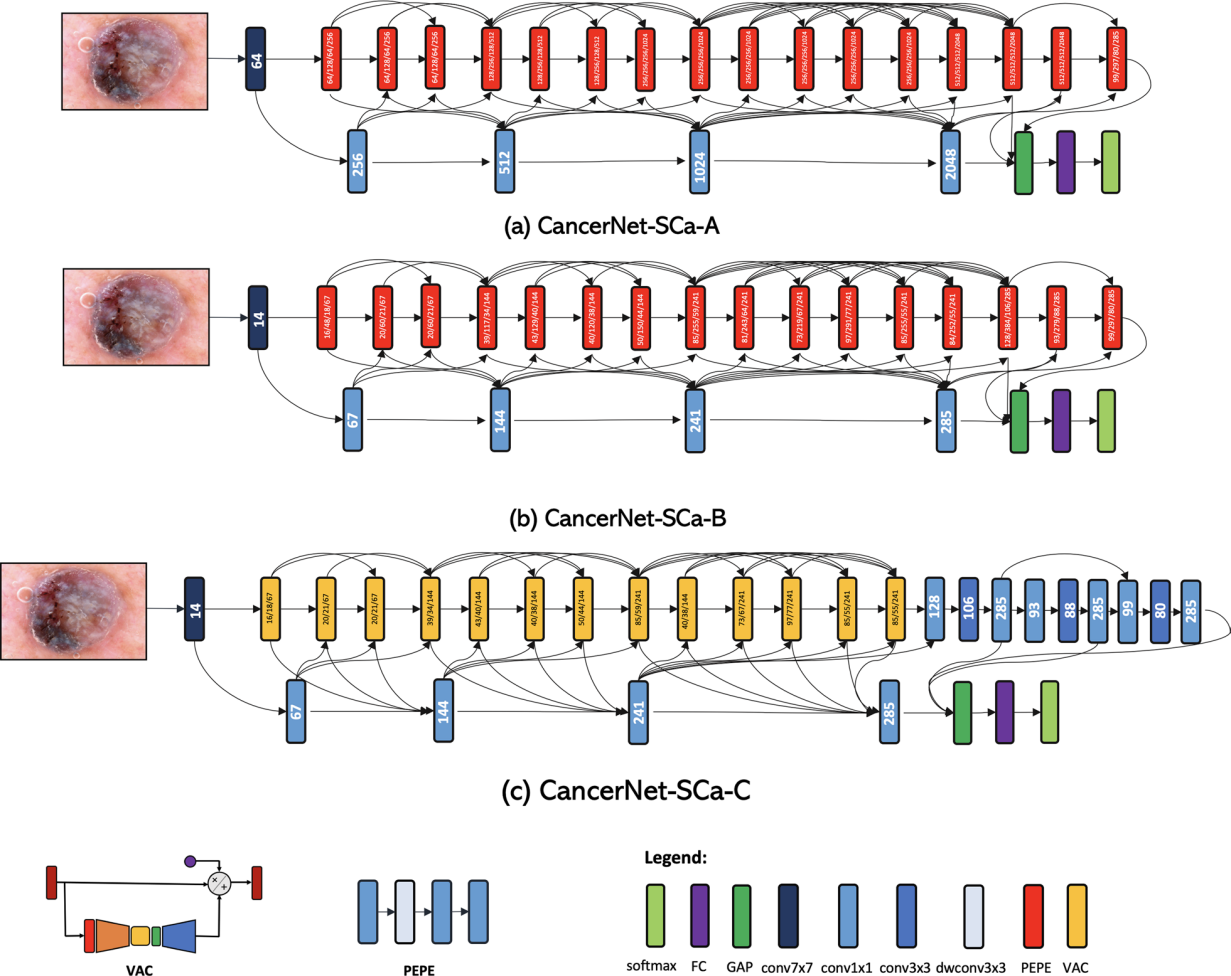
The proposed Cancer-Net SCa network architectures. The number in each convolution module represents the number of channels. The numbers in each visual attention condenser represents the number of channels for the down-mixing layer, the embedding structure, and the up-mixing layer, respectively (details can be found in [15]). It can be observed that all Cancer-Net SCa architectures exhibit both great macroarchitecture and microarchitecture design diversity, with certain models exhibiting specific lightweight macroarchitecture design characteristics such as attention condenser and projection–expansion–projection–expansion (PEPE) design patterns comprised of channel dimensionality reduction, depthwise convolutions, and pointwise convolutions

The proposed Cancer-Net SCa architectures are shown in Fig. 2. A number of interesting observations can be made about the Cancer-Net SCa architectures, which illustrates the efficacy of leveraging machine-driven design for constructing highly customized deep neural network architectures tailored for skin cancer detection from dermoscopy images, as opposed to leveraging pre-existing, pre-defined deep neural network architecture designs in existing literature. Even though the Cancer-Net design leverages residual architecture design principles, the underlying macroarchitecture and microarchitecture designs are substantially different than that of standard ResNet designs in numerous aspects that provide substantially better accuracy as well as efficiency.

#### Diverse, heterogeneous designs

First of all, it can be observed that the macroarchitecture designs exhibited in all Cancer-Net SCa networks are highly diverse and heterogeneous, with a mix of spatial convolutions, pointwise convolutions, and depthwise convolutions, all with different microarchitecture designs. In contrast, the macroarchitecture design of ResNet is largely homogeneous and consists of a uniform sequence of spatial convolutions and pointwise convolutions with repeated microarchitecture designs within each stage. In addition, the designs of all Cancer-Net SCa network architectures leverages depthwise convolutions for greater computational and architectural efficiency while achieving strong representational capacity, while the ResNet architecture design does not.

Furthermore, it can be observed that the macroarchitecture design of Cancer-Net SCa-A is drastically different than that of Cancer-Net SCa-C, with the majority of components in the two deep neural network architectures leveraging different design patterns. These two observations reflect the fact that a machine-driven design exploration strategy was leveraged and allows for very fine-grained design decisions to be made to best tailor for the task of skin cancer detection. To craft such diverse, highly customized macroarchitecture and microarchitecture designs for the different Cancer-Net SCa models by hand would not be possible, and speaks to the great potential for high customization via a machine-driven design exploration strategy.

#### Lightweight design patterns

Second, it can be observed that very lightweight design patterns are exhibited in the proposed Cancer-Net SCa architectures. For example, Cancer-Net SCa-A and Cancer-Net SCa-B both leverage projection–expansion–projection–expansion (PEPE) design patterns comprised of channel dimensionality reduction, depthwise convolutions, and pointwise convolutions. The PEPE module was discovered by a machine driven exploration strategy [15], and comprises of a projection layer that reduces dimensionality via pointwise convolution, followed by an expansion layer that increases dimensionality via depthwise convolution, followed by another projection layer and another expansion layer sequentially. Additionally, PEPE modules leverage channel decoupling by splitting the input channels from the previous layer into separate partitions of channels, where each partition is then processed independently of each other. The particular sequential combination of dimensionality reduction, channel decoupling, and pointwise feature mixing enables a strong balance between representational capacity and representational efficiency, thus resulting in highly efficient yet effective deep convolutional neural network architectures.

In another example, light-weight long-range connectivity patterns can be observed, which enable improved representational power by enabling information from earlier layers to flow directly to later layers while maintaining connection efficiency by only leveraging it sparingly.

#### Efficient self-attention mechanisms

Third, it can be observed that Cancer-Net SCa-C exhibits a highly efficient self-attention architecture design that leverages the concept of visual attention condensers (VAC) [14, 15]. In comparison, the ResNet architecture does not include these VAC modules that allow the Cancer-Net models to achieve far greater efficiency while achieving selective attention. The improvements that these allow are shown in both the network performance and efficiency, and can be seen in Tables 1 and 2.

**Table 1:** Comparison of parameters, FLOPs, and accuracy for tested network architectures on the ISIC dataset

Paper	Architecture	Parameters (M)	FLOPs (G)	Accuracy (%)
Budhiman et al. [19]	ResNet-50 [38]	23.52	7.72	78.3
Demir et al. [20]	Inception V3 [18]	23.80	43.6	84.2
Hassan et al. [58]	DenseNet-121 [57]	7.00	2.80	83.9
Ech-Cherif et al. [59]	MobileNetV2 [45]	4.20	0.57	83.9
	Cancer-Net SCa-A	13.65	4.66	83.7
	Cancer-Net SCa-B	**0**.**80**	0.43	**84**.**4**
	Cancer-Net SCa-C	1.19	**0**.**40**	83.9

Best results highlighted in bold

**Table 2:** Sensitivity, positive predictive value (PPV), and negative predictive value (NPV) comparison on the ISIC dataset

Architecture	Sensitivity (%)	PPV (%)	NPV (%)
ResNet-50 [38]	78.7	78.0	78.5
Inception V3 [18]	76.9	**89**.**9**	79.8
DenseNet-121 [57]	80.5	86.4	81.8
MobileNetV2 [45]	84.6	83.5	84.4
Cancer-Net SCa-A	**92**.**8**	78.5	**91**.**2**
Cancer-Net SCa-B	91.4	80.2	90.0
Cancer-Net SCa-C	90.0	80.2	88.7

Best results highlighted in bold

The visual attention condensers that are used in the Cancer-Net design are efficient self-attention mechanisms that produce condensed embeddings characterizing joint local and cross-channel activation relationships, and perform selective attention accordingly to improve representational capability. The utilization of attention condensers enable greater attentional performance and efficiency within the deep neural network architecture without the typical increase in computational overhead imposed by other previously proposed self-attention mechanisms for visual perception [54, 55]. The VAC module consists of a down-mixing layer, condensation layer, an embedding structure, an expansion layer, a selective attention mechanism, and an up-mixing layer, in that order [15]. The initial down-mixing layer and condensation layers learn and project the input activations to a reduced dimensionality with an emphasis on strong activation proximity, in order to maintain powerful feature extraction despite having a more condensed representation. The output is embedded using the embedding structure, then projected to an increased dimensionality via the expansion layer, thus producing self-attention values that well represent the relevant regions of interest. Next, the input, the self attention values, and the self-attention mechanisms are multiplied and the product is projected back to the original channel dimensionality for the final output. The addition of the learned mixing layers into the attention condenser design enables a better balance between joint spatial-channel embedding dimensionality and selective attention performance, which we leverage to create the highly efficient Cancer-Net SCa designs. The leveraging of lightweight design patterns is important as it better facilitates for real-time diagnosis, potentially on digital dermoscopy scanners. The unique designs of Cancer-Net SCa thus illustrate the benefits of leveraging machine-driven design exploration to create deep neural network architectures tailored to skin cancer detection.

### Explainability-driven performance validation

To audit Cancer-Net SCa in a responsible and transparent manner, we take inspiration from [25] and conducted an explainability-driven performance validation by leveraging GSInquire [56], an explainability method that has been shown to provide state-of-the-art quantitative interpretability performance in a way that reflects the decision-making process of the underlying deep neural network. The leveraging of explainability for performance validation serves several important purposes.

#### Behavioural validation

The transparency gained through explainability allows us to ensure that decisions made by Cancer-Net SCa are not based on erroneous visual cues, but on clinically relevant visual indicators as captured in the dermoscopy image. Any abnormal behaviour in the decision-making process of the deep neural networks, such as gaps and biases as well as ’right decision, wrong reason’ scenarios can then be identified during this performance validation process and corrected in the appropriate way.

#### Insight discovery

The ability to understand the visual indicators used in the decision-making process of Cancer-Net SCa when predicting whether a skin lesion is cancerous or not can enable dermatologists and research scientists to gain better insight into what visual cues may be important for detecting skin cancer. The discovery of such potentially valuable visual indicators may be interesting and shed new light on improved clinical screening strategies based on such visual indicators.

#### Building clinical trust

Given that the ultimate purpose of the proposed Cancer-Net SCa deep neural network architectures is to facilitate for computer-aided clinical decision support, it is crucial for the widespread adoption of such deep neural networks to provide explainability and interpretability to dermatologists. This improves the level of trust they can place on the additional information provided by the neural network, and augments their own knowledge and experience with the model.

## Results and discussion

In this section, we will present the experimental results to evaluate the efficacy of the proposed Cancer-Net SCa deep neural network architectures for skin cancer detection from dermoscopy images. We conduct two forms of analysis for a more comprehensive understanding of the proposed networks. First, we conduct empirical evaluation using the ISIC dataset and evaluate the proposed deep neural network architectures using a suite of quantitative performance metrics, and compare them against three other popular network architectures seen in literature. Next, we conduct a qualitative analysis using an explainability-driven performance validation to better understand the decision-making behaviour and validate the relevance of the imaging features learned from the ISIC dataset. The details and discussion of each experiment is presented below.

### Quantitative results

We evaluate the performance of the proposed Cancer-Net SCa deep neural network designs using the test set of dermoscopy images described in “Data preparation” section. Furthermore, for evaluation purposes, we compare it with the 50-layer residual deep convolutional neural network architecture [17], which was leveraged by Budhiman et al. [19] to achieve the best quantitative results in their experiments. In addition, an Inception V3 [18] network was used for comparison, which was leveraged in several studies [20, 21] for strong quantitative results in their experiments. These commonly used ResNet-50 and Inception architectures were chosen due to the fact that they actually demonstrated superior, state-of-the-art performance on this dataset when compared to more complex architectures [19–21]. For additional comparisons against commonly used architectures, as well as to compare against more lightweight designs, we also evaluated the performance against DenseNet-121 [57], which was leveraged by Hassan et al. [58] to achieve strong performances in the skin lesion classification domain, and MobileNetV2 [45], which was used by Ech-Cherif et al. [59] to develop a mobile application for skin cancer classification on edge devices.

The performance metrics leveraged in this study are accuracy, sensitivity, positive predictive value (PPV), and negative predictive value (NPV). Construction and evaluation are conducted using TensorFlow [60], with tested architectures trained using Adam optimizer, with LR=0.0001, epochs=80, momentum=0.9, and batch rebalancing. The usage of batch rebalancing helps alleviate the major imbalances in the training and validation datasets.

The results are shown in Tables 1, 2 and 3. A number of observations can be made. First, it can be observed that the proposed Cancer-Net SCa designs achieved comparable accuracy when compared to the four traditionally used network architectures while achieving significantly reduced architectural and computational complexity. For example, Cancer-Net SCa-B achieved 0.7% higher accuracy when compared to the ResNet-50 network architecture while possessing $29.5{\times }$ fewer parameters and requiring $\sim 18\times$ fewer FLOPs. This illustrates the benefits of leveraging a machine-driven design exploration strategy for designing a deep neural network architecture that finds a strong balance between efficiency and accuracy. When compared against the Inception V3 network architecture, the Cancer-Net SCa-B design attains comparable accuracy while having significantly lower computational complexity (by one or two orders of magnitude), with Cancer-Net SCa variants A and C also having orders of magnitude lower architectural complexity while maintaining comparable accuracy. Further literature can also be compared against, such as [61] also suggesting lightweight architectures for similar use cases, proposing small and efficient networks with just 4.8M parameters, but Cancer-Net SCa-C is still just a quarter of the size while maintaining competitive accuracy. As such, this makes the Cancer-Net SCa designs much more well-suited for on-device skin cancer screening when compared to other tested network architectures.

**Table 3:** Confusion matrix breakdown for Cancer-Net SCa-A

	Predicted	
	Benign (%)	Malignant (%)
*Label*		
Benign	**74**.**66**	25.34
Malignant	7.24	**92**.**76**

Higher is better, indicated in bold

Second, it can be observed that all proposed Cancer-Net SCa designs achieved higher sensitivity and negative predictive value (PPV) than that achieved with the other tested deep neural network architectures used for comparison. For example, Cancer-Net SCa-A achieved 14.1% higher sensitivity and 10.8% higher NPV when compared to the ResNet-50 network architecture. Compared to the most sensitive deep neural network tested (MobileNetV2), Cancer-Net SCa A, B, and C achieved 9.6%, 8.0% and 6.4% higher sensitivity, respectively. This further illustrates the benefits of leveraging a machine-driven design exploration strategy for designing a highly customized deep neural network architecture tailored specifically for skin cancer detection. Although the Cancer-Net SCa networks achieved lower PPV than the architectures tested, the significantly higher sensitivity values are generally more valuable in the clinical workflow for skin cancer screening, where false negatives should be avoided as much as possible.

Observing the CancerNet variants alone, we can learn that the Cancer-Net SCa designs have different performance-efficiency tradeoffs, with Cancer-Net SCa-A providing the highest sensitivity and NPV, Cancer-Net SCa-B having the lowest architectural complexity, highest accuracy and highest PPV between all Cancer-Nets, and Cancer-Net SCa-C having the lowest computational complexity. This illustrates how a machine-driven design exploration strategy can allow for greater flexibility to meet the requirements of the use case (e.g., on-device examination vs. cloud-driven examination).

### Qualitative results

**Fig. 3:**
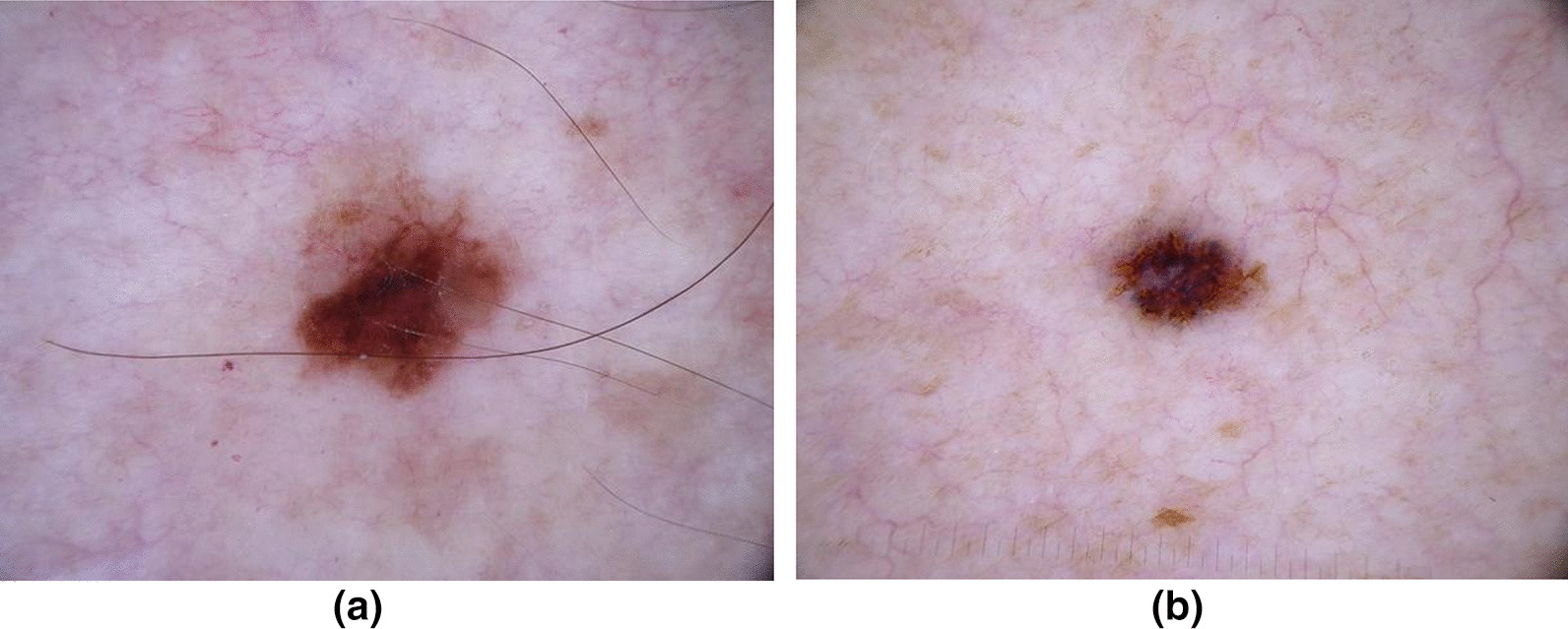
Sample images from the ISIC Dataset that the trained models were tested on. All seven tested model architectures correctly identified image **a** as malignant. However, the DenseNet and Inception architectures incorrectly classified image **b** as benign, while all three Cancer-Net SCa models had a correct classification of malignant

Visual comparisons of the images themselves are also useful when analysing model performance, to try and gain insights on which images were classified correctly and incorrectly. Figure 3 shows two sample images from the ISIC dataset, which are relatively similar in appearance. All seven tested model architectures correctly identified image (a) as malignant, while the DenseNet-121 and InceptionV3 architectures incorrectly classified image (b) as benign. All three Cancer-Net SCa models had a correct classification of malignant for both images. However, to fully understand the differences between models, a purely metric based analysis is insufficient when understanding the reasons behind superior performance.

**Fig. 4:**
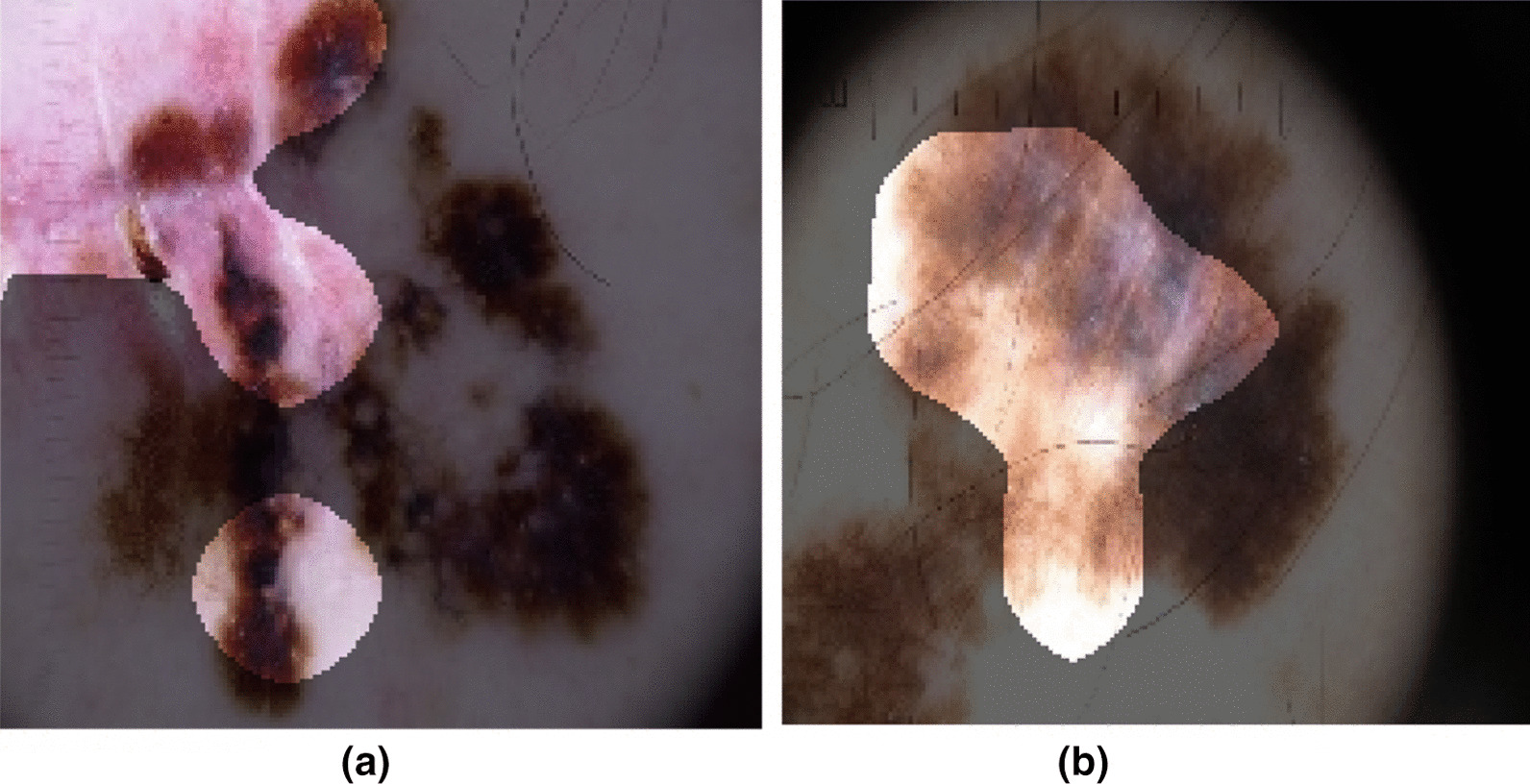
Example dermoscopy images of malignant cases from the ISIC dataset and their associated diagnostically relevant imaging features as identified by GSInquire [56], using Cancer-Net SCa-A. The bright regions indicate the imaging features identified to be relevant

**Fig. 5:**
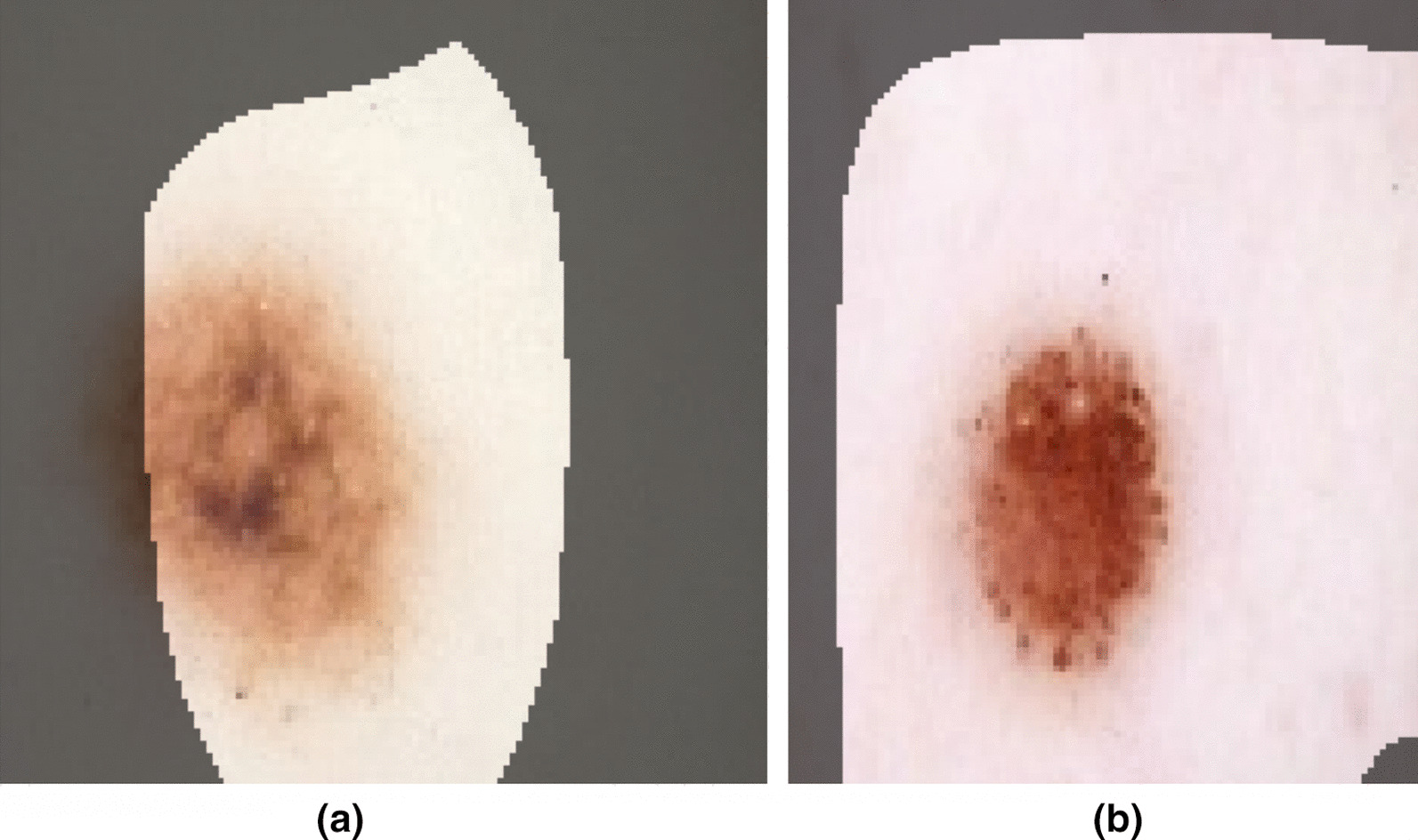
Example dermoscopy images of benign cases from the ISIC dataset and their associated diagnostically relevant imaging features as identified by GSInquire [56], using Cancer-Net SCa-A. The bright regions indicate the imaging features identified to be relevant

In order to better understand how Cancer-Net SCa makes detection decisions based on dermoscopy images, we leveraged GSInquire [56] for explainability-driven performance validation and insight discovery to audit the decision-making process. Examples of dermoscopy images of malignant cases and benign cases and the associated imaging features identified by GSInquire to be relevant to the decision-making process of Cancer-Net SCa-A are shown in Figs. 4 and 5.

A number of observations can be seen based on the identified imaging features that provide key insights into the decision-making behaviour of Cancer-Net SCa-A. First of all, it can be observed that Cancer-Net SCa-A leverages the color heterogeneities within a skin lesion to aid in the differentiation between benign and malignant skin lesions (as exemplified in both Fig. 4a, b). Second, it can also be observed that the textural heterogeneity in a skin lesion aids in the differentiation between benign and malignant skin lesions (as exemplified in Fig. 4b). Third, it can be further observed that morphological irregularities exhibited by skin lesions is leveraged by Cancer-Net SCa-A to differentiate between benign and malignant skin lesions (as exemplified in both Fig. 4a). Fourth, it can be also observed from Fig. 5 that, in the case of benign lesions the morphological symmetry and relative homogeneous textural characteristics of the entire skin lesion and surrounding skin area are leveraged by Cancer-Net SCa-A to identify that these are benign cases. Therefore, these types of diagnostically relevant imaging features that Cancer-Net SCa-A leverages indicate that the deep neural network is exhibiting valid decision-making behaviour.

As such, it can be clearly seen that an explainability-driven performance validation helps screen for erroneous decision-making behaviour that rely on irrelevant visual indicators and imaging artifacts. This emphasizes the importance of auditing deep neural networks when designing for clinical applications as it can increase the trust that practitioners have towards deep learning.

### Discussion and broader impact

Skin cancer continues to be a prominent problem for the health and well-being of society, with millions of new cases and thousands of deaths annually costing billions of dollars in the United States alone. Not only are most biopsies unnecessary (only 1 in 36 biopsies yield a case of melanoma [62]), the cost of misdiagnoses and unnecessary biopsies can quickly accumulate. This adds an expensive and needless burden to both the patient and the system, while taking up precious time which could be allocated to treating additional patients.

The benefits of computer-aided strategies such as Cancer-Net SCa are twofold. Not only do they provide dermatologists with valuable second opinions during diagnosis, they also save time by acting as pre-screening tools in the diagnostic process. The goal of Cancer-Net SCa is not to replace dermatologists, but instead to aid professionals in their decision-making processes as well as act as a basis for others to improve upon and accelerate advances in this area. When correctly leveraged with professional knowledge, Cancer-Net SCa will hopefully impact the field of dermatology in a positive manner. The fact that Cancer-Net SCa underwent explainability-driven auditing will hopefully allow for greater trust as well as better understanding of its decision-making behaviour. As one of the major deterrents of deep learning in the medical field is the “black-box” nature of these systems, granting additional insight on how network decisions are reached can result in more trust towards the systems - a crucial first step towards the widespread adoption of artificial intelligence for health and safety.

To reiterate, these Cancer-Net SCa designs were tailor made for rapid computer-aided clinical decision support on edge and embedded devices (such as portable digital dermoscopy systems or low-cost mobile smartphones), or other resource-limited environments (such as dermoscopes attached to low-cost, low-power tablets and laptops).

## Conclusion

In this study, we introduced Cancer-Net SCa, a suite of deep neural network designs tailored for the detection of skin cancer from dermoscopy images, each with a different balance in performance and efficiency. Designed via a machine-driven design exploration strategy, Cancer-Net SCa is available open source and available to the general public. Experimental results using the ISIC dataset show that the proposed Cancer-Net SCa designs can achieve strong skin cancer detection performance while providing a strong balance between computational and architectural efficiency and accuracy. An explainability-driven audit of Cancer-Net SCa is also conducted, showing that prediction is performed by leveraging relevant abnormalities found within skin lesion images, rather than random visual indicators and imaging artifacts.

Given the promise of leveraging a machine-driven design exploration strategy for creating highly customized deep neural network architectures for skin cancer detection, we aim to explore and expand upon this strategy by creating deep neural networks tailored to other forms of cancer such as lung cancer, breast cancer, and prostate cancer. The strategy can also extend to different clinical decision support tasks such as risk stratification, treatment planning, and therapy response prediction for improved personalized patient care. Furthermore, we aim to continue making these tailored deep neural networks along with associated scripts and curated benchmark datasets publicly available in open source and open access form for researchers, clinicians, and citizen data scientists alike to leverage and build upon them to advance research and adoption in this area.

## Data Availability

This work is publicly available at https://github.com/jamesrenhoulee/CancerNet-SCa. Training and inference scripts are provided, along with a suite of pre-trained models. The datasets generated and analysed during the current study are available at https://www.isic-archive.com/.
